# Are the tools fit for purpose? Network inference algorithms evaluated on a simulated lipidomics network

**DOI:** 10.1093/bioadv/vbaf286

**Published:** 2025-11-09

**Authors:** Finn Archinuk, Haley Greenyer, Ulrike Stege, Steffany A L Bennett, Miroslava Cuperlovic-Culf, Hosna Jabbari

**Affiliations:** Department of Biomedical Engineering, University of Alberta, Edmonton, Alberta T6G 1H9, Canada; Department of Computer Science, University of Victoria, Victoria, BC V8P 5C2, Canada; Department of Computer Science, University of Victoria, Victoria, BC V8P 5C2, Canada; Neurolipidomics Laboratory, Department of Biochemistry, Microbiology and Immunology, University of Ottawa, Ottawa, ON K1H 8M5, Canada; Department of Chemistry and Biomolecular Sciences, University of Ottawa, Ottawa, ON K1N 6N5, Canada; Department of Cellular and Molecular Medicine, University of Ottawa, Ottawa, ON K1H 8M5, Canada; Neurolipidomics Laboratory, Department of Biochemistry, Microbiology and Immunology, University of Ottawa, Ottawa, ON K1H 8M5, Canada; Department of Chemistry and Biomolecular Sciences, University of Ottawa, Ottawa, ON K1N 6N5, Canada; Department of Cellular and Molecular Medicine, University of Ottawa, Ottawa, ON K1H 8M5, Canada; Digital Technologies Research Center, National Research Council of Canada, Ottawa, ON K1K 4P7, Canada; Department of Biomedical Engineering, University of Alberta, Edmonton, Alberta T6G 1H9, Canada

## Abstract

**Motivation:**

Various methods have been proposed to construct metabolic networks from metabolomic data; however, small sample sizes, multiple confounding factors, the presence of indirect interactions as well as randomness in metabolic processes are of major concern.

**Results:**

In this study, we benchmark existing algorithms for creating correlation- and regression-based networks of changes in metabolite abundance and evaluate their performance across different sample sizes of a generative model. Using standard interaction-level tests and network-scale analyses based on centrality scores, we assess how well these methods recover represented metabolomic networks. Our findings reveal significant challenges in network inference and result interpretation, even when sample sizes are significant and data are the result of computer modeling of metabolic pathways. Despite these limitations, we demonstrate that correlation-based network inference can, to some extent, discriminate between two different metabolic states in the computational model. This suggests potential utility in distinguishing overarching changes in metabolic processes but not direct pathways in different conditions.

**Availability and implementation:**

All relevant data is provided at https://github.com/TheCOBRALab/metabolicRelationships

## 1 Introduction

The metabolome encompasses all small molecules within a system, and can be profiled using various mass spectrometry and nuclear magnetic resonance “omics” techniques (Wishart 2007). Advances in these techniques have enabled new approaches to understanding disease, increasingly integrating the analysis of individual metabolite abundances into metabolic networks (Wilkins and Trushina 2018). Metabolomic networks can be formulated as graphs, where the nodes represent metabolites and the edges connecting them indicating some level of covariation (Liu *et al.* 2020). These metabolic networks direct experimental exploration of molecular pathways or identify changes resulting from pathological conditions (Szymanski *et al.* 2009).

“Network inference” is commonly used to deduce edges from metabolic concentration data. However, this process is often limited by (i) the small number of reference samples relative to the many metabolites and their potential interactions (Nyamundanda *et al.* 2013), and (ii) the difficulty of distinguishing direct from indirect interactions across broad, rapidly changing concentration ranges. These challenges are further compounded by the need to separate truly metabolic relationships from signaling or other regulatory associations. Network inference algorithms (NIAs) often rely on simplifying assumptions, such as linear relationships, steady-state conditions, and sparsity of interactions, to make computations feasible. However, these assumptions often fail to capture the complexities of biological systems, which are dynamic, nonlinear, and subject to experimental noise. Additionally, inferred connections may reflect correlations rather than true causal relationships, emphasizing the need for careful interpretation and experimental validation.

In hybrid, data- and knowledge-driven network reconstruction, prior information about metabolic pathways and reactions is used to prioritize edges that match known biochemistry (Cuperlovic-Culf *et al.* 2023). These methods can help assess the significance of established reactions in a given system, but they inherently bias the analysis toward known interactions and still depend on the accuracy of the data-driven inference algorithm.

This paper addresses issues of network inference by extending the work of Jahagirdar *et al.* (2019) to better understand how (i) evaluation metrics change with small sample sizes, (ii) investigate where errors occur in inferred networks, and (iii) establish how inferred networks can be used to differentiate metabolitic states. We find that network inference based exclusively on pairwise interactions between metabolites fails to accurately capture the biological network. These deficits are not simply due to a lack of samples, rather they demonstrate a failure to converge to the reality of the underlying network. This work adds to the growing movement to examine the assumptions baked into pairwise NIAs (Lingjærde *et al.* 2020, Peel *et al.* 2022, Li *et al.* 2024).

Our first goal is to determine which evaluation metrics best indicate when an inferred network closely approximates a reference network. To that end, we introduce an *aspirational network*: starting from the true adjacency matrix, we inject controlled noise to isolate the effect of sample size while removing confounding from biological outliers and network inference algorithm (NIA)-specific limitations. Although there is no exact mapping from sample size to signal quality, we assume signal quality increases with the number of samples.

Next, we move beyond pairwise edge accuracy to evaluate the *structure* of the network. We use graph-theoretic centrality measures as a natural way to assess overall inference quality, with particular attention to connectivity of terminal and central nodes. Because all inferred networks share the same node set, we can score each node’s connectivity against centrality values computed from the ground-truth network.

Finally, after having demonstrated that NIAs do not converge to the underlying interaction network, we test whether inferred networks could be used to differentiate biological conditions—another important research area in metabolomics. We adapted our simulation model to examine arachidonic acid (AA) lipidomics in sarcopenia, an age-dependent skeletal muscle-wasting syndrome. Guided by the data and conclusions of Palla *et al.* (2021), we compared the ability of correlation- and regression-based network approaches to recover the sarcopenia-associated enzymatic change implied by those data. Palla *et al.* used high-performance liquid chromatography tandem mass spectrometry (LC-MS/MS) to measure prostaglandin family members in young and aged murine skeletal muscle and then identified the causal enzymatic expression changes responsible for measured age-dependent differences in prostaglandin abundances. They found that increased expression of 15-hydroxyprostaglandin dehydrogenase (15-PGDH) in aged tissue contributed to skeletal muscle atrophy. In our network, 15-PGDH enzyme catalyzes two reactions: Prostaglandin E2 (PGE2) $\overset{}{\to }$ 15-keto-PGE2, and prostaglandin D2 (PGD2)$\overset{}{\to }$ 15-keto-PGD2. Palla *et al.* showed that reductions in PGE2 and PGD2 levels in aged tissue result from increased degradation to 15-keto-PGE2 and 15-keto-PGD2, respectively. They further demonstrated that the resulting loss of PGE2-associated signaling, in part, causes sarcopenia-related muscle wasting.

## 2 Materials and methods

### 2.1 Simulated metabolic network

**Figure 1.**
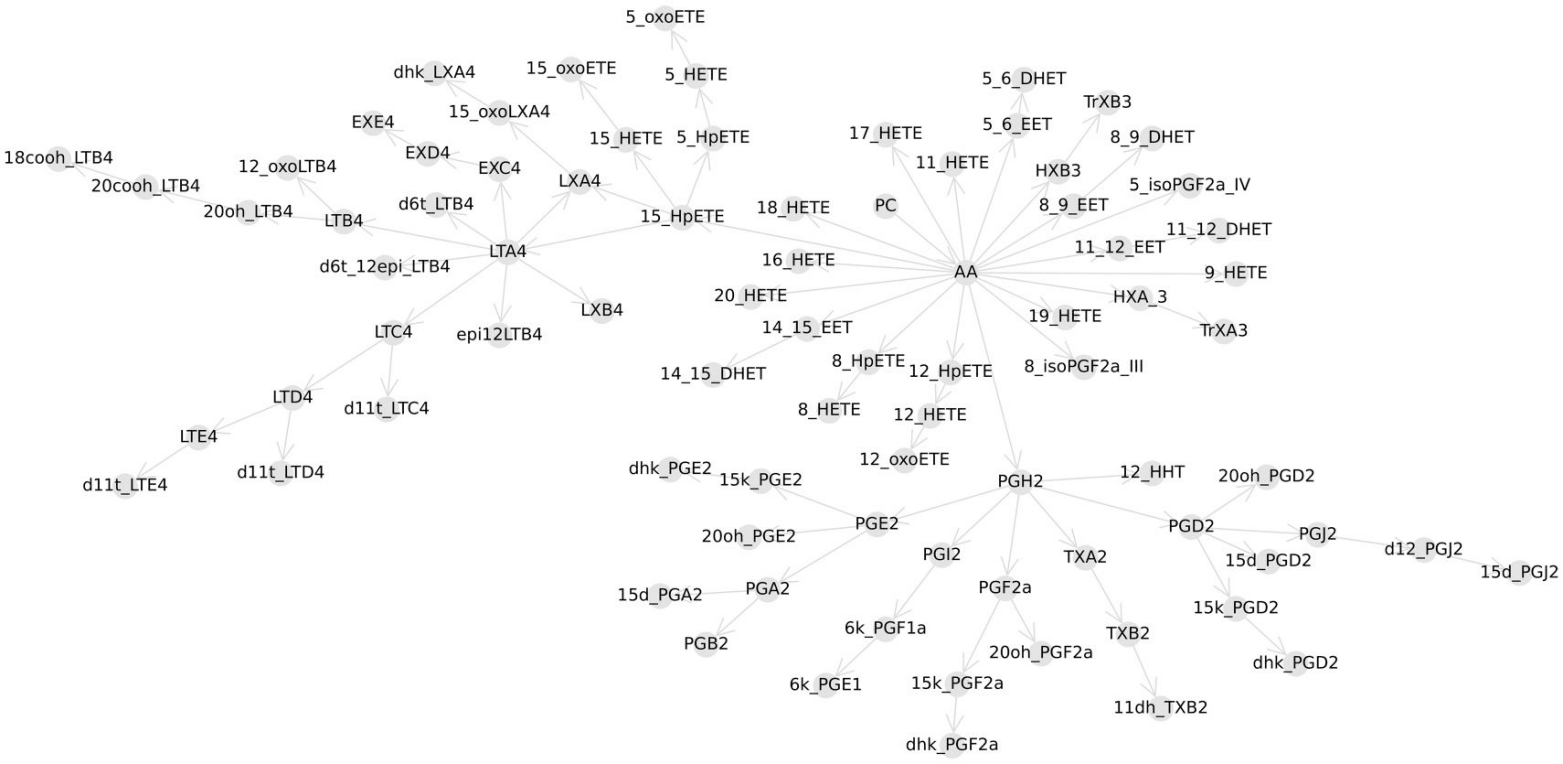
The simulated Arachidonic acid network representing primary interactions. Nodes represent metabolites. Connections between nodes indicate a direct reactionary interaction, either through enzymatic conversion or mass action.

We used the computational model developed by Jahagirdar *et al.* of AA degradation and benchmarked this model against several NIAs (Jahagirdar *et al.* 2019). The simulated network was constructed based on known reactions involved in AA metabolism. The constructed AA metabolic model included 83 metabolites and 131 reactions. Reactions were written as ordinary differential equations, with enzymatic reactions described using Michaelis-Menten kinetics and non-enzymatic mass action laws (Michaelis *et al.* 2011). Figure 1 shows the interactions between metabolites. Edges indicate a reaction in the AA model; removal of metabolites from the system are not shown but are present at each terminal node. The network aggregates multiple metabolites under the umbrella of phosphatidylcholine (PC). This simplification is secondary to the focus of our work of evaluating network inference algorithms against a biologically inspired metabolomic ground truth.

The adjacency matrix for the AA model provides information about the presence or absence of reactions. This is a n×n matrix, where *n* represents the number of nodes (i.e. metabolites) in the network. Interactions between two nodes are indicated by a “1” in the corresponding row and column of the matrix if there exists a reaction, otherwise “0” indicates no interaction. The simulated network generates samples by initializing reaction parameters and running the model forward until steady state is reached. By randomizing initial reaction parameters, we created samples, each in the form of a vector: (a,b,…,z) where *a*, *b*, … and *z* are metabolite concentrations measured at the end of the simulation.

### 2.2 Evaluation metrics

In its simplest form, all possible pairs of metabolite interactions can be organized into a matrix (*A*) representing all potential interactions. It is assumed that weaker interactions indicate pairs that are more distant in the network. These interactions were quantified using an association function:


(1) $$A_{x,y}=\text{Assoc}(x,y)$$

where for each entry $A_{x,y}$, *x* and *y* represent a given pair of metabolites, and Assoc() represents an association function produced by a given NIA. When a threshold τ is applied to the entries of *A*, a binary matrix *B* is obtained:


(2) $$B_{x,y}=\{\begin{array}{c} 1\;\;\text{if}\;A(x,y)>\tau \\ 0\;\;\text{otherwise} \end{array}$$

with τ being a fixed value, typically 0.6 for correlation networks (Suarez-Diez and Saccenti 2015). When applicable, the absolute value is used, particularly in correlation-based methods, where strong negative correlations are also considered significant. To remove the influence of tuning a threshold value, we dynamically selected a threshold for each inferred network to maximize the F1-score. The F1-score is calculated as the harmonic mean of precision and recall:


(3) $$F1=\frac{2\text{TP}}{2\text{TP}+\text{FP}+\text{FN}}$$

where TP, FP, and FN indicate number of true positives, false positives, and true negatives, respectively. Since biological networks are often sparse and therefore imbalanced, we also tested the optimal threshold using the Matthews Correlation Coefficient (MCC) (Matthews 1975):


(4) $$\text{MCC}=\frac{\text{TP}\times \text{TN-FP}\times \text{FN}}{\sqrt{\left(\text{TP}+\text{FP})(\text{TP}+\text{FN})(\text{TN}+\text{FP})(\text{TN}+\text{FN}\right)}}$$

Different metrics capture distinct aspects of the inferred network, and no single evaluation metric is sufficient to fully assess the quality of an NIA. The evaluation metrics we considered fall into two categories: pairwise interactions evaluate the network using edge-level comparisons to the reference network and centrality measures evaluate longer range connectivity.

### 2.3 Pairwise interaction measures

Area under the receiver operating characteristic curve (AUROC) and area under the precision-recall curve (AUPR) are evaluation methods that assess performance by scanning across all possible threshold values applied to association matrices, generating curves that balance two desirable properties.

The ROC curve reflects the trade-off between the True Positive Rate (TPR=TP/(TP+FN)) and the False Positive Rate (FPR=FP/(TN+FP)). The TPR represents the proportion of true positive classifications correctly captured in the predicted network. The FPR indicates the proportion of false positives included. Ideally, the TPR is 1, and the FPR is 0.

AUROC is a widely used metric for evaluating classification performance, especially in diagnostic tests. While effective at distinguishing between “bad” and “good” models, AUROC has been shown to lack sensitivity in differentiating among “good” models (Marzban 2004). Additionally, AUROC performs poorly on imbalanced datasets, such as sparse binary networks commonly encountered in metabolomics (Boughorbel *et al.* 2017).

As a complement to ROC, Precision-Recall (PR) is widely used for evaluating binary classifiers. PR reflects the trade-off between the Positive Predictive Value (PPV, or precision) and the True Positive Rate (TPR, or recall). The PR curve is constructed similarly to the ROC curve, replacing TPR and FPR with PPV and TPR, respectively.

AUPR provides a single summary statistic of classifier performance. Given the imbalanced nature of binary biological networks, where non-edges greatly outnumber edges, AUPR is increasingly preferred over AUROC for assessing network inference algorithms in bioinformatics (Saito and Rehmsmeier 2015).

MCC (Matthews 1975) is widely used for binary classification, particularly for evaluating network inference methods on imbalanced data, where it often outperforms AUROC (Boughorbel *et al.* 2017). MCC quantifies the correlation between reference and predicted classifications, with values ranging from −1 to 1 (Matthews 1975). A value of +1 indicates perfect prediction, −1 indicates complete disagreement, and 0 suggests performance equivalent to random classification.

### 2.4 Centrality measures

Four centrality measures were selected to summarize the inferred networks, informed by prior work (Liu *et al.* 2020). Degree centrality measures the number of connections a node has, with higher values typically indicating greater importance in the network. This measure is normalized by the total number of nodes. Many biological networks, including metabolic networks, exhibit a scale-free structure, where node degrees follow a power-law distribution. This results in a network with many weakly connected nodes and a few highly connected hubs (Liu *et al.* 2020).

Betweenness centrality assesses a node’s influence on the flow of communication between other nodes, highlighting those that serve as critical intermediaries (Freeman 1977). The betweenness centrality of a given node ($v_{i}$) is defined as:


(5) $$\text{BC}(i)=\sum_{v_{j},v_{k}\in V,j\neq k}\frac{\delta_{j,k}(i)}{\delta_{j,k}}$$

where *V* is the set of network nodes, $\delta_{j,k}$ is the number of the shortest paths from nodes *j* to *k* and $\delta_{j,k}(i)$ is the number of these shortest paths that pass through node $v_{i}$.

Closeness centrality measures the importance of a node based on its average distance to all other reachable nodes in the network. Nodes with higher closeness centrality can propagate information more efficiently, requiring fewer hops to reach other nodes. The closeness centrality of a given node $v_{i}$ can be calculated as the mean value of the inverse of the distance from $v_{i}$ to other nodes:


(6) $$\text{CC}(i)=\frac{1}{N-1}\sum_{v_{j}\in V,j\neq i}\frac{1}{d_{ij}}$$

where *N* is the total number of nodes in the network, and the distance between two nodes, $v_{i}$ and $v_{j}$, $d_{ij}$ is defined as the shortest path (minimum number of edges to be traversed) between them (Liu *et al.* 2020).

PageRank centrality (*R*) measures the importance of a node based on the importance of its neighbors. Values are iteratively updated over *t* iterations or until convergence, as defined by the following equation:


(7) $$R_{i}(t)=\alpha \sum_{j=1}^{N}A{\text{dj}}_{ij}\frac{R_{j}(t-1)}{\text{deg}(j)}+(1-\alpha )\frac{1}{N}$$

where α is the probability of being influenced by a neighboring node, ${\text{Adj}}_{ij}$ indicates the presence of an edge between nodes *i* and *j*, and deg(j) is the degree of node *j* (Liu *et al.* 2020).

PageRank is primarily designed for directed graphs but has been adapted for undirected graphs by treating each undirected edge as bidirectional. PageRank guarantees convergence and avoids degenerate behavior that can arise in graphs with disconnected components (Liu *et al.* 2020).

Centrality measures were calculated for each node in the reference network and compared to those from the inferred network. Differences were summarized by mean absolute error (MAE) and repeated 100× to show a confidence interval of 95%. A perfectly recovered network will have a residual error of 0.

### 2.5 Aspirational network

We define the aspirational network with a signal level ranging from 0 to 1. At all signal levels, the association matrix was initialized as a uniformly distributed random matrix with values between 0 and 1 ($U_{\left[0,\;1\right]}$). The signal level adjusts the matrix by incorporating a proportion of the ground-truth interacting edges:


(8) $$\text{Asp}(x,y,\text{signal})=U_{\left[0,\;1\right]}+\text{signal}\times \text{Adj}(x,y)$$

where Adj(x,y) is 1 if an interaction exists between metabolites *x* and *y* in the reference adjacency matrix, and 0 otherwise. The resulting association matrix values are strictly positive and range from 0 to 2. Fifty signal levels between 0 and 1 were selected with increments of 0.02. For each signal level 100 aspirational association matrices were generated to provide information about variance.

## 3 Network inference algorithms evaluated and their assumptions

### 3.1 Correlation-based methods

Correlation-based methods are widely used in network inference due to their computational efficiency and scalability for large datasets (Saint-Antoine and Singh 2020). These methods quantify pairwise associations between metabolites, offering a simple and intuitive approach to constructing networks. However, a key limitation is their inability to distinguish between direct and indirect interactions, often leading to system-wide correlations that obscure the true network structure (Krumsiek *et al.* 2011).

**Pearson’s correlation** (${\text{CORR}}_{P}$) is one of the most common correlation-based methods. It assumes a normal data distribution and identifies linear relationships between variables (Pearson 1920). The correlation coefficient ranges from −1 to 1, with ${\text{CORR}}_{P}(x,y)=\pm 1$ indicating perfect linear dependence and ${\text{CORR}}_{P}(x,y)=0$ indicating complete linear independence. While its simplicity is appealing, Saccenti *et al.* (2019) highlight that measurement errors bias correlation coefficients toward zero, potentially impacting the significance of inferred edges.

**Spearman’s correlation** (${\text{CORR}}_{S}$) can be considered an extension of ${\text{CORR}}_{P}$ wherein the data is converted to ranks before calculating the coefficient (Spearman 1904). Spearman’s correlation makes no assumptions of the underlying data distribution and can detect both monotonic and linear relationships. Similar to Pearson’s correlation coefficient, a value of ${\text{CORR}}_{S}(x,y)=\pm 1$ indicates a perfect monotonic relationship between *x* and *y* while ${\text{CORR}}_{S}(x,y)=0$ indicates monotonic independence. Since biological systems are governed by non-linear interactions, Spearman correlation is preferred given sufficient sample size (Camacho *et al.* 2005).

**Kendall’s correlation** (${\text{CORR}}_{K}$) is similar to Spearman’s correlation, in that it measures the strength of associations based on the relative ordering of data (Kendall 1938). Kendall’s correlation is less sensitive to outliers and provides more reliable *P* values with smaller sample sizes, making it advantageous in these contexts.

**Biweight midcorrelation** (bicor) is designed to be more robust to outliers compared to ${\text{CORR}}_{P}$, which is particularly valuable when working with small sample sizes. The bicor method has been successfully applied in areas such as gene regulatory network inference and differential coexpression analysis (Zheng *et al.* 2014). Similar to other correlation measures, bicor(x,y) produces coefficients ranging from ±1, with 0 indicating independence.

**Partial correlation** (pcor) quantifies the relationship between two variables after accounting for their linear dependencies on other variables, making it useful for distinguishing direct from indirect associations. Partial correlation has been used in metabolic network inference but are computationally expensive and require a larger number of samples (Krumsiek *et al.* 2011).

Previous studies have provided key insights into the expected performance of correlation methods in different situations. ${\text{CORR}}_{S}$ performs well in metabolomic studies due to its reliance on monotonicity rather than linearity given a sufficient number of samples. When sample sizes are limited, it has been recommended to use ${\text{CORR}}_{P}$ with a high significance threshold (Camacho *et al.* 2005).

Moreover, de Siqueira Santos *et al.* (2014) have evaluated the strengths of various correlation methods using both simulated and experimental gene expression data. Their findings suggest that when monotonicity can be assumed, ${\text{CORR}}_{S}$ or ${\text{CORR}}_{K}$ are preferable to ${\text{CORR}}_{P}$, as they can detect both linear and non-linear monotonic relationships with greater statistical power.

### 3.2 Information theoretic-based methods

Information theoretic methods use **mutual information** (MI) to find pairwise scores for interactions. Similar to correlation-based methods, MI can be used on its own to produce weighted networks or with thresholding to produce binary networks (Butte and Kohane 1999). The mutual information MI(x,y) between two metabolites *x* and *y* can be calculated as (Song *et al.* 2012):


(9) $$\text{MI}(x,y)=\sum_{i,j}^{n}p(x_{i},y_{j})\;\text{log}\;\frac{p(x_{i},y_{j})}{p(x_{i})p(y_{j})}$$

where $p(x_{i},y_{j})$ is the joint probability distribution function of $x_{i}$ and $y_{j}$ and $p(x_{i})$ and $p(y_{j})$ are the probabilities that $x=x_{i}$ and $y=y_{i}$, respectively. Entries of an association matrix generated using MI can have values in the range of [0,+∞]. MI presents a theoretical advantage over correlation-based approaches by capturing non-linear relationships, which are known to be present in biological systems (Camacho *et al.* 2005). However, it is important to note that recovering non-linear relationships with limited samples is challenging, and estimating MI from small sample sizes introduces significant errors (Song *et al.* 2012).

The **Context Likelihood of Relatedness** (CLR) algorithm was designed as an extension of MI (Faith *et al.* 2007). The CLR algorithm estimates the likelihood of the MI score for a given pair of metabolites against the background of the MI scores for that pair, minimizing the effect of indirect interactions. CLR can be used with any association matrix as a post-processing step.

**MRNET** evaluates an MI association matrix for inference based on the maximum relevance/minimum redundancy (MRMR) algorithm (Ding and Peng 2005, Meyer *et al.* 2007). The MRMR algorithm iteratively selects a feature that has maximum relevance with respect to the target variable and minimum redundancy relative to the features that have been selected in previous iterations (Ding and Peng 2005). As a feature selection technique, MRMR has been successfully applied to disease biomarker identification (Mahendran *et al.* 2021). In its original publication, MRNET was shown to perform competitively against CLR on 30 simulated microarray data sets (Meyer *et al.* 2007).

### 3.3 Regression-based methods

Regression-based methods quantify relationships between metabolites by solving regression problems to predict a target metabolite based on others. These methods can infer potential causal relationships (Saint-Antoine and Singh 2020) and several have been benchmarked on DREAM5 (Marbach *et al.* 2012).

**Gene Network Inference with Ensemble of Trees** (GENIE3) uses variable selection with ensembles of regression trees to infer associations. Its tree-based approach allows GENIE3 to detect non-linear relationships (Huynh-Thu *et al.* 2010). Briefly, biological samples are represented as vectors, with features corresponding to metabolites. To determine associations for a target metabolite *x*, *x* is masked, and regression trees are trained to predict it based on the remaining metabolites. Metabolites most important for predicting the masked values are identified as being highly associated with *x*. The ensemble of trees ranks the importance of each input metabolite. For consistency with other methods used in this work, directionality was removed by taking the maximum of $A_{x,y}$ and $A_{y,x}$.

### 3.4 Resampling-based methods

The **Probabilistic Context Likelihood of Relatedness of Correlation** (PCLRC) algorithm extends CLR by iteratively resampling subsets of the available data. In each iteration, the top *Q*% of highest-associated metabolite interactions are recorded. The final association matrix reflects the fraction of times each metabolite pair appeared in the top *Q*% interactions.

Following the original procedures (Saccenti *et al.* 2015, Jahagirdar *et al.* 2019), each resampling used 75% of the available data, and the top 30% of interactions were saved. Although $10^{4}$ repetitions were originally recommended, here we limited the number to $10^{3}$ due to the computational demands of applying PCLRC across multiple association matrices.

### 3.5 Computational settings

Five hundred synthetic samples were generated from the simulated AA model using MATLAB (R2022a). Each NIA utilized subsets of these samples with sizes n=[5,10,20,50,100,200]. For each method and sample size, 100 repetitions were conducted allowing us to calculate a 95% confidence interval. We included subsets of size 100 and 200 to evaluate the NIAs when the number of samples was greater than the number of metabolites.

We followed the approach laid out in Jahagirdar *et al.* (2019) to ensure we reached steady state by running the simulation for 90 h. As with Jahagirdar *et al.* variation between profiles was achieved by slightly perturbing the reaction parameters via uniformly resampling from ±10% of the parameters’ optimized values. Association matrices were generated for each algorithm from the following sources: SciPy (Virtanen *et al.* 2020) provided Kendall-Tau and Spearman correlations. The Python version of GENIE3 (Huynh-Thu *et al.* 2010) was obtained from Github. Numpy had the most optimized Pearson correlation algorithm in terms of wall-clock time and core utilization (Harris *et al.* 2020). Biweight midcorrelation was rewritten from Pingouin to speed up the algorithm by compilation (Vallat 2018). MRNET was rewritten due to challenges with implementing the official source in this workflow. CLR and PCLRC are processes applied to an association matrix, and were rewritten to allow flexibility in this workflow. Partial Correlation was performed with the Python package Pandas (McKinney 2010). Sci-kit Learn (Pedregosa *et al.* 2011) was used for calculating MI by way of entropy with k-nearest neighbours, where *k* defaults to 3. The rewritten algorithms were accelerated using Numba compilation (Lam *et al.* 2015). Code is available at https://github.com/TheCOBRALab/metabolicRelationships, and data is available at https://doi.org/10.5281/zenodo.17246740.

### 3.6 Simulated sarcopenia

To simulate the young and aged states in prostaglandin abundances in our network, we started from the reaction parameter values reported by Jahagirdar *et al.* (2019) and amplified $V_{\text{max}}$ by 1000×, informed both by the increase in 15-PGDH mRNA observed by Palla *et al.* (2021) and prior work correlating mRNA to protein levels (Lahtvee *et al.* 2017, Buccitelli and Selbach 2020). $K_{m}$ was also increased by 2.5× for the two affected reactions. We generated 200 samples from these simulated young and aged networks to obtain the largest sample sizes considered in this study. Each sample was initialized from a steady-state. Enzyme parameters were updated using the ±10% method, then simulated for 20 hours to reach their new equilibrium using COPASI (Hoops *et al.* 2006).

### 3.7 Bootstrapped network differentiation

To find differences between the aged and young inferred networks, we took inspiration from PCLRC to minimize the impact of sample selection. To create the young network we had a pool of 200 samples from the young distribution. We randomly pulled 100 samples (with replacement) to create a network using ${\text{CLR}}_{P}$ NIA. This was repeated 100× to create 100 networks. A final network was created by selecting the edges that occurred reliably (i.e. in >50% of networks). This was repeated for the aged data samples to create a final aged network.

As a quick investigation of whether this resulted in reliable edges, we split the 100 networks into two groups and compared the difference in reliable edges.

## 4 Results

### 4.1 Performance measure traits

We used the aspirational network (see Section 2.5) to control signal levels, allowing us to investigate how evaluation measures are affected by noise.

Consistent with previous work demonstrating that AUROC does not utilize the full metric range (0–1) (Marzban 2004), we found that values saturated early (i.e. at relatively low signal level; Fig. 1A, available as supplementary data at *Bioinformatics Advances* online), indicating AUROC was unable to differentiate between multiple “good” networks. Our data also confirmed that AUPR values were saturated at both signal-to-noise extremes (Fig. 1B, available as supplementary data at *Bioinformatics Advances* online). Both area under curve metrics showed low variance across signal-to-noise levels (Fig. 1A and B, available as supplementary data at *Bioinformatics Advances* online).

FDR has low variance for the majority of signal-to-noise levels (Fig. 1C, available as supplementary data at *Bioinformatics Advances* online). MCC has been shown to perform well for low signal-to-noise networks, does not exhibit discontinuities, and has nearly uniform variance across the signal range (see Section 2.2). Here, there was a slight saturation of the measure at high signal (Fig. 1D, available as supplementary data at *Bioinformatics Advances* online). Using FDR and MCC, we compared thresholds chosen by maximizing either F1-score or MCC and found only minor differences. Accordingly, we use F1-score to set thresholds in subsequent analyses. Visualizations of all pairwise evaluation metrics are available in the Supplementary Material (Fig. 1, available as supplementary data at *Bioinformatics Advances* online).

**Figure 2.**
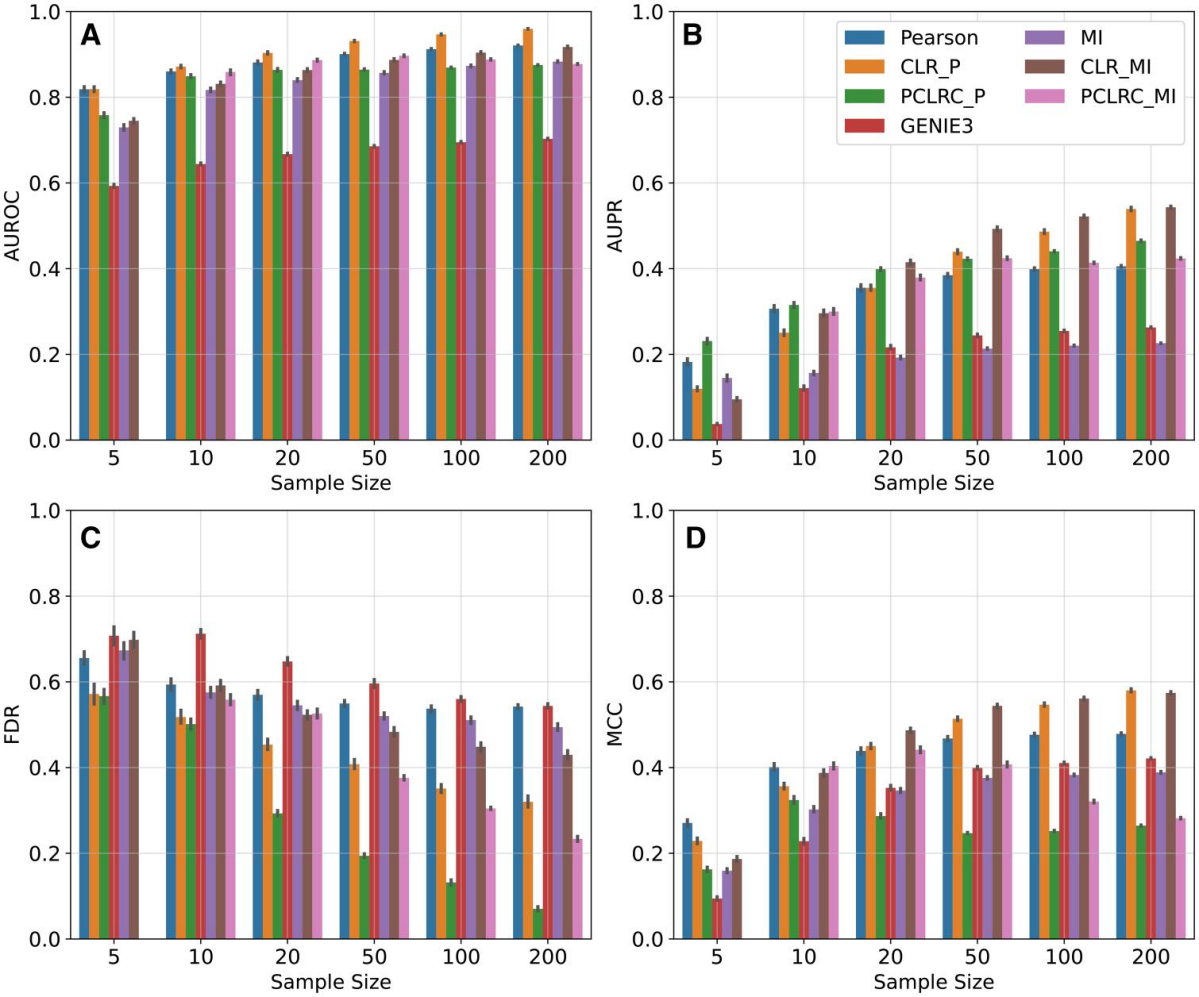
Pairwise metric evaluations for a selection of Network Inference Algorithms. (A] AUROC. (B] AUPR. For (C] FDR and (D] MCC a threshold was selected that optimized for F1-score to ensure the best possible result for cross-evaluation.

Centrality evaluation measures were also inspected for their behavior using the aspirational network algorithm with varying signal levels (Fig. 2, available as supplementary data at *Bioinformatics Advances* online). Betweenness centrality had a discontinuity in the curve, likely resulting from biologically incorrect edges bringing metabolites together that should be apart in the low signal regime. Properly recovered edges began to appear at higher signals (>0.6). Closeness centrality showed a stark discontinuity at low signal that we again attributed to random edges confusing this metric. While degree centrality starts with high variance, it quickly converged to a low error with signal >0.2, making it potentially useful for differentiating NIAs during evaluation and development. The PageRank performed well across the signal ranges >0.2, with a nearly linear relationship to signal.

### 4.2 Network inference evaluation

The previous section provided insight into how various performance measures were affected by noise. It is commonly assumed that additional samples will reduce noise and thus reveal the signal required to infer a metabolic network (Suarez-Diez and Saccenti 2015). Our results show that this assumption is not relevant for reasonable experimental sample sizes, and that even large sample sizes do not converge to reveal the underlying network structure.

### 4.3 Pairwise measures

We tested NIAs across sample sizes using the pairwise evaluation measures outlined above. Figure 2 shows a subset of NIAs for visual clarity the full set of NIAs tested are available in Supplementary Materials, available as supplementary data at *Bioinformatics Advances* online. ${\text{PCLRC}}_{\text{MI}}$ was omitted from the five sample size evaluations due to MI requiring at least three samples and PCLRC selecting a subset of those available. As discussed above, AUROC (Fig. 2A) began to converge for higher sample sizes at approximately 0.9 for most methods. As expected, we observe an increase in AUPR as the number of samples increases (Fig. 2B). Post-processing methods (PCLRC and CLR) typically achieve higher AUPR over the raw-association methods. The highest AUPR achieved was from CLR methods at 200 samples, though no method reached an AUPR of >0.6 (Fig. 2B). As sample size increased, the FDR (Fig. 2C) declined—indicating fewer incorrect edges—and post-processing NIAs typically accelerated this trend. The low variance of FDR at these very small sample sizes suggested that the high variance at low signal is an artifact that can be ignored. PCLRC methods are much better able to remove outlier influence with larger sample sizes than the corresponding raw method (≥50 samples); however, these approaches provide only marginal improvement with low sample sizes (≥10) (Fig. 2D). For NIAs that use the raw association output, FDR plateaued at 0.5. Finally, using MCC, the NIA models without post-processing showed a plateau at 0.5, that was approached early and was not overcome (Fig. 2D). ${\text{CORR}}_{P}$ performed best at low sample sizes (≤10). PCLRC methods peaked at sample sizes around 20 and decreased with more samples. This surprising result was further explored in Supplementary Materials: PCLRC Algorithmic Limit, available as supplementary data at *Bioinformatics Advances* online. We show that this early evaluation peak appeared to be caused by saving only the top interactions for each resampling. NIAs using CLR were the only methods that crossed 0.5 for this metric, and only when the number of samples was very high (≥50, Fig. 2D). As MCC is designed to avoid the challenges of imbalanced data, failing to perform well here shows that none of these NIAs appreciate the sparse connectivity of the underlying network.

### 4.4 Centrality analysis

**Figure 3.**
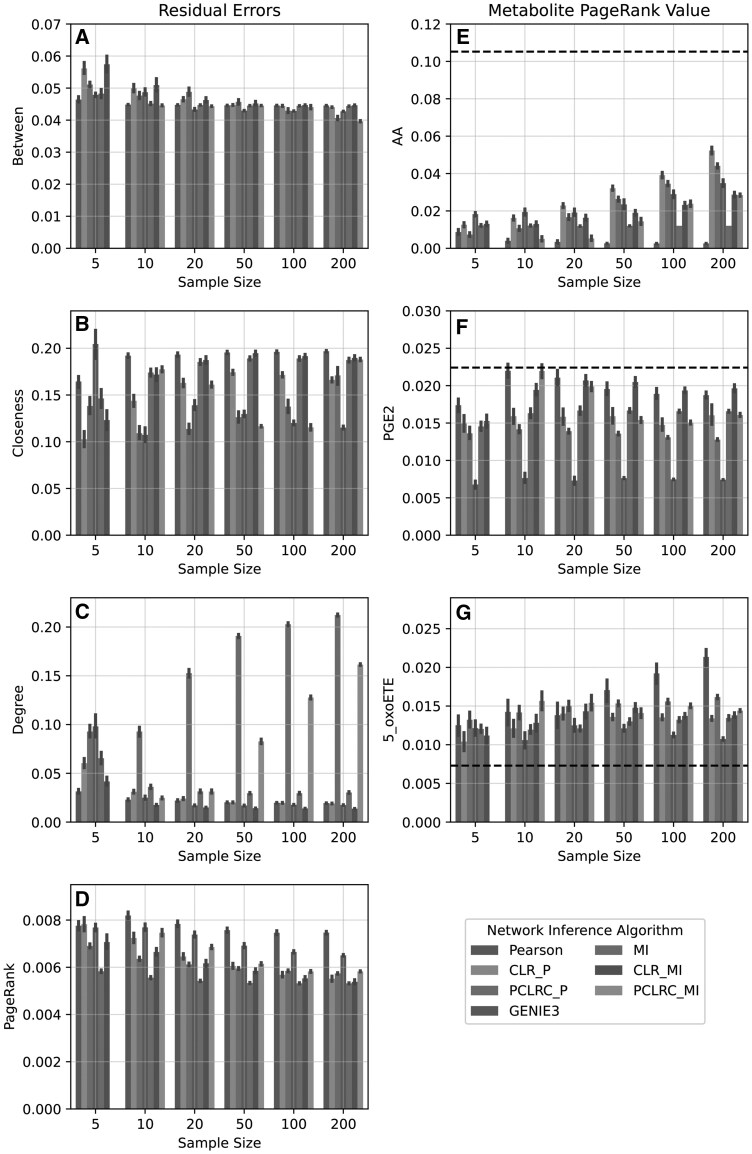
Centrality metrics for a selection of NIAs. (A–D) Differences between the centrality vector from inferred networks and the reference network. Zero indicates the inferred network matches the reference network. (E–G) PageRank centrality for individual metabolites: AA [core metabolites], PGE2 [intermediate in the network], and 5-oxoETE [distal metabolites]. The black line indicates the PageRank value of these metabolites in the reference network.

Centrality-based evaluation measures compare the centrality score of the metabolite in the inferred network against the centrality score of the metabolite in the underlying network. Figure 3 shows the average error (left column) tends to decrease with additional samples (as expected, but with notable exceptions).

Betweenness showed marginal improvement with additional samples regardless of NIA method (Fig. 3A). PCLRC methods showed a drop at 200 samples, but the overall trend converged to a different point from the reference network. Closeness (Fig. 3B) had high variance between runs, and many methods appeared to be diverging from the reference network as the number of samples increased. Degree centrality (Fig. 3C) indicated that PCLRC methods diverge with additional samples. PageRank showed limited improvement with additional samples (Fig. 3D). MI had the lowest PageRank error at 200 samples, but the error was comparable to five samples.

### 4.5 Systematic PageRank error

**Figure 4.**
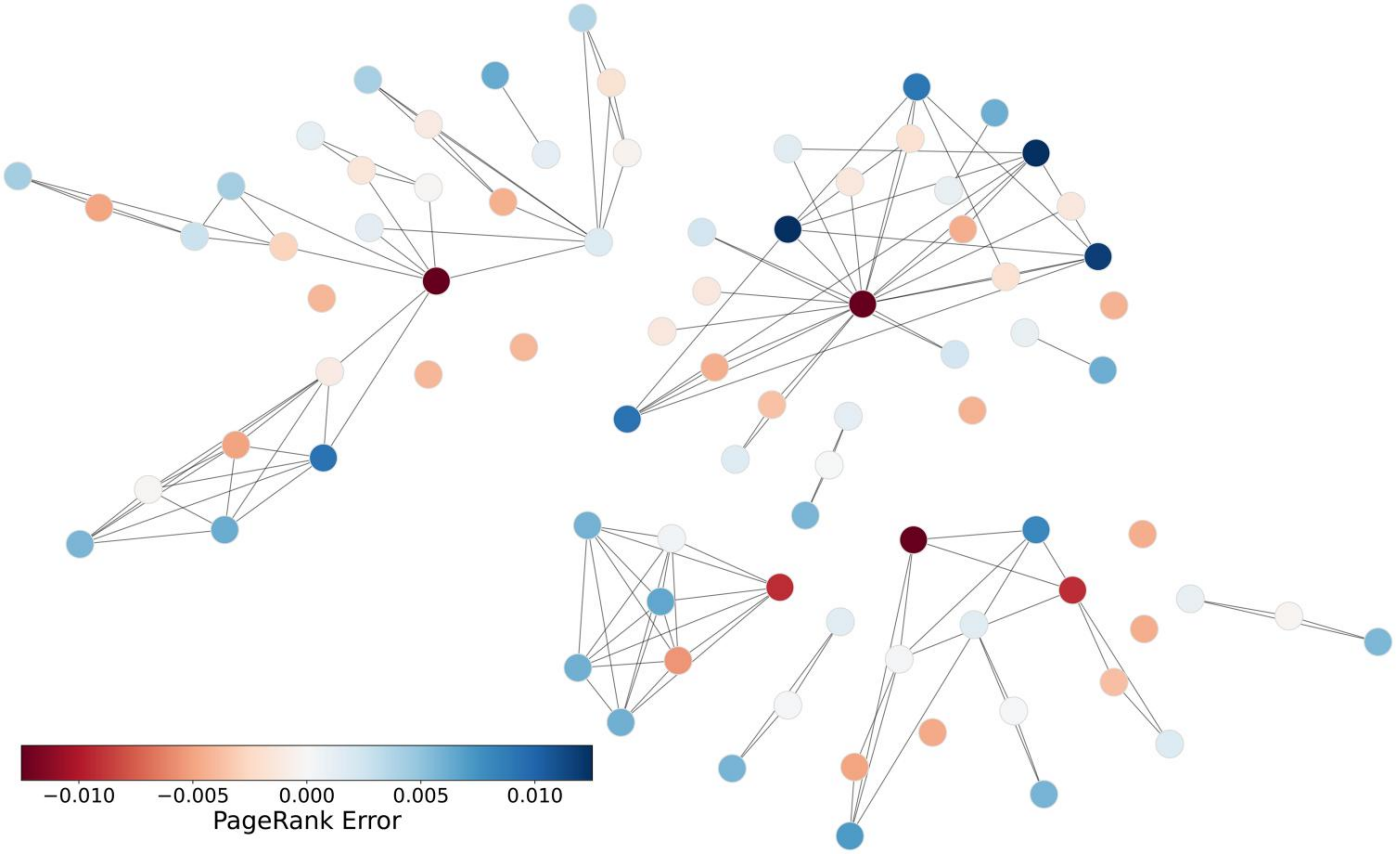
A single example of a recovered network using ${\text{CLR}}_{P}$ inference with 200 samples. Nodes are colored by their disagreement with the PageRank value calculated for the reference network, with red indicating underestimated values and blue indicating overestimated values.

We next assessed where errors accumulated across the networks. We isolated three metabolites at different points in the reference network: AA representing a central node, PGE2 representing an intermediate node, and 5-oxoETE representing a distal node. Figure 3E–G separates these metabolites by sample size with the dotted black line indicating the PageRank value of that node in the reference network. The PageRank of the corresponding metabolite was calculated across 100 repetitions for each of the tested NIAs. AA (Fig. 3E) was consistently underestimated in importance across methods. PGE2 (Fig. 3F) was rarely estimated correctly; note the large error bars and the lack of convergence even as sample size increased. By contrast, 5-oxoETE (Fig. 3G) was overestimated by all methods. Figure 4 visualizes the PageRank error of each metabolite by their position in the AA network. These values were inferred using ${\text{CLR}}_{P}$ with 200 samples. This representation clearly demonstrates that nodes towards the exterior of the network were systematically overestimated while highly central nodes were underestimated.

### 4.6 Bootstrapped networks

We created two networks based on our simulated sarcopenia modification of the AA network (detail in Materials and Methods for the modification and the network creation). By comparing the inferred networks to those from the same source distributions we found ${\text{CLR}}_{P}$ was reliably recovering edges. We found four edges in disagreement and 112 in agreement in the young network, and five edges in disagreement and 127 in agreement in the aged network. Across states, 38 edges were differentially inferred (Supplementary Fig. 5C, available as supplementary data at *Bioinformatics Advances* online).

**Figure 5.**
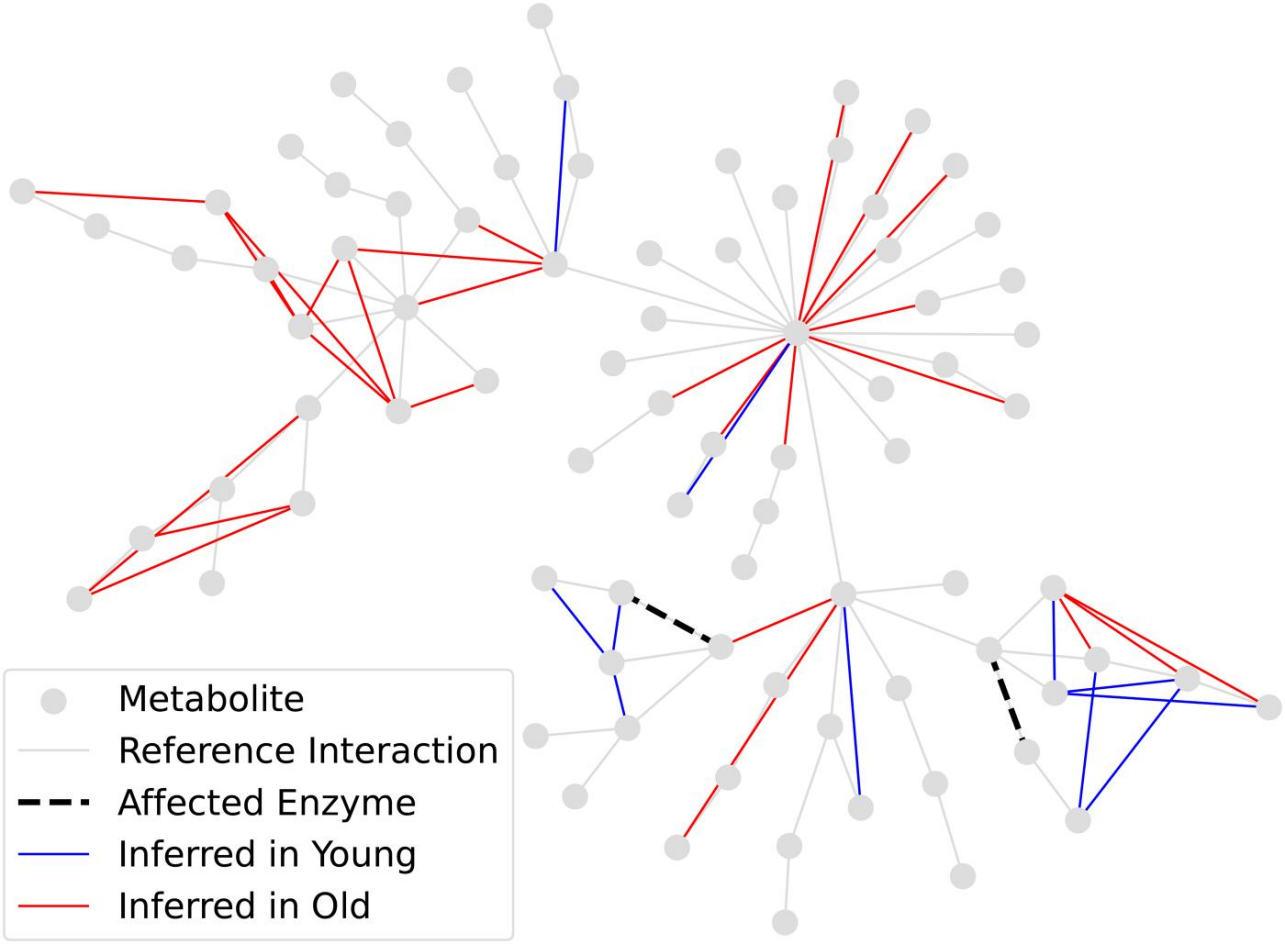
Edges that are in disagreement between the young and aged distributions. Gray lines depict the interactions defined by the reference network between metabolites (gray nodes). Black dotted lines show the two 15-PGDH enzyme reactions. Blue lines indicate edges that were regularly found in the young network, but not the aged network, whereas red lines indicate the opposite. Note that the *bona fide* enzymatic disruption identified by Palla *et al.* (2021) was not identified as a disagreement between the young and aged networks.

Figure 5 visualizes edges in disagreement across distributions. Edges marked in blue were found in the young but not aged distribution. Red edges were present in aged but not young distributions. These visualizations show that the edges directly affected by the 15-PGDH enzyme (black dotted lines) were not detected by this differential analysis, and the resulting disagreement is not confined to neighboring reactions. Notably, these modified reactions were inferred in both the young and aged networks.

## 5 Discussion

We evaluated NIAs using a simulated dataset because it offers several advantages over biological data. Simulations provide a known ground-truth network, enabling precise, objective assessment of algorithm performance—including error identification and accuracy quantification. By contrast, lipidomic datasets typically lack a validated interaction network (Cuperlovic-Culf *et al.* 2023), making it difficult to separate true, direct biological interactions from algorithmic artifacts. Simulated data also allow controlled experiments by systematically varying factors such as sample size and noise, while avoiding complications like metabolic compartmentalization. Using a simulated AA degradation network, we show that NIAs—without additional prior knowledge or constraints—fail to recover the underlying metabolic network.

We started by characterizing the limits of network evaluation measures by introducing an aspirational network that allowed us to control the total noise. The output of any NIA is limited by the number of samples (analogous to the fraction of adjacency matrix signal), and therefore “aspires” to the aspirational network given the assumption that the NIA will converge to the underlying metabolic network. Our aspirational network is parallel to Diez *et al.* (2015), in which the authors looked at the number of samples required for network convergence. However, their focus was convergence to the inferred network when sample size is not limiting, whereas we assess convergence toward the underlying network (which they did not have access to). We found MCC and PageRank performed well across signal levels and collectively inform the user about both edge-level and network-level quality. Preferring MCC over AUROC for evaluation is also supported by prior work (Chicco and Jurman 2023).

We tested several popular NIAs that find pairwise associations between metabolite concentrations to infer the underlying network using varying sample sizes. Determining the quality of the inferred networks relied on both edge-level and network-level evaluation measures. We found that while the edge-level evaluation measures often improved with additional samples (AUPR, AUROC, FDR), there were also algorithmic limitations (MCC; see Supplementary PCLRC Algorithmic Limit, available as supplementary data at *Bioinformatics Advances* online). Additional samples caused the evaluation metrics to converge, but not to the expected optimal values of the metric. These evaluation measures were under the optimal threshold value, requiring knowledge of the ground-truth interaction network. We chose this idealized methodology to focus on the best-case scenario for each NIA method. As the NIAs we evaluated are primarily correlation-based (with the notable exception of GENIE3), they predict undirected networks. By relaxing the directionality of the reference network edges, our evaluations are a further simplification in network inference.

The centrality-based evaluation measures more directly demonstrate NIAs do not converge to the underlying network, even across almost 2 orders of magnitude of samples. The surprising stability of centrality measures across sample sizes brought us to interrogate where NIAs were introducing errors. We found PageRank values for central metabolites were systematically underestimated while distal metabolites were overestimated. PageRank was originally designed for directed graphs. It is important to note that while the underlying network is directed, the inferred networks are not. We found a key challenge with centrality-based evaluation measures is the sensitivity to errant edges. For instance, a well-reconstructed network with a single additional edge between distant metabolites can significantly increase closeness values compared to the ground truth.

While our work has not identified what biological relationships NIAs exactly capture, it does add *in silico* examples to the increasing number of calls that a better understanding of the underlying utility of network inference is needed (Nyamundanda *et al.* 2013). In cases where the underlying metabolic network is known, alternative hybrid methods have shown interesting results (Li *et al.* 2024); however, this type of information is not always available.

We do show that, although inferred networks do not recover the underlying metabolic interactions well, they can be used to differentiate disease states. Caution should be applied when inferring candidate reactions responsible for this change of state since the impacted reactions can be dispersed in the network, far from the causative reaction/mutation/expression. This utility (and deficit) was clearly demonstrated by simulating age-associated changes in lipid metabolism in sarcopenia informed by experimental results of a previous study (Palla *et al.* 2021). A change of state between young and aged networks was identified but the empirically validated causal reaction could not be inferred. We inspected the perturbed reactions directly by enumerating the presence of the edges across the 100 repetitions. Prostaglandin E2 (PGE2) $\overset{}{\to }$ 15-keto-PGE2, was inferred in 96 aged networks, and in all young networks. Prostaglandin D2 (PGD2)$\overset{}{\to }$ 15-keto-PGD2 was inferred in 99 repetitions in the aged networks and 85× in the young networks. Since these edges are highly reliable regardless of the source, network differentiation by edge difference was unable to identify the causative reaction.

This work focused on network inference using small sample sizes of exclusively concentration data. Understanding how to make the most of this type of data is important for maximizing the value of available datasets, especially given the increase in these datasets and the parallelizability of correlation-based approaches for larger systems. However, our results suggest that without additional constraints no amount of samples will be sufficient for network inference. Incorporating other data modalities can provide additional insight into network structure while relying on mass-spectroscopy derived features. For example, Amara *et al.* (2022) propose creating networks of metabolite mass differences, structural similarities through fragment analysis, and isotopic grouping. Each network would provide additional insight into which interactions are plausible without needing *a priori* knowledge of the network.

## Supplementary Material

vbaf286_Supplementary_Data

## Data Availability

All relevant data is provided at https://github.com/TheCOBRALab/metabolicRelationships.
