# Supplementary material for: Are the tools fit for purpose? Network inference algorithms evaluated on a simulated lipidomics network
Source: Bioinform Adv. 2025 Nov 9;5(1):vbaf286. doi: 10.1093/bioadv/vbaf286 (PMC12640239; doi:10.1093/bioadv/vbaf286)
Supplement: vbaf286_Supplementary_Data [file vbaf286_supplementary_data.pdf]

Supplementary Materials

Performance Measure Characterization

We characterized how these evaluation metrics performed under an ideal scenario to account for the additional challenges of sample size error and NIA biases. We defined a 'best-case scenario' inference algorithm in Section *Aspirational Network* and we show here the pairwise evaluations in Supplementary Figure S1. For FDR and MCC, each association matrix had a threshold selected to optimize F1-score to create a binarized network. We also used using MCC to determine an optimal threshold and found minimal disagreement when converging to the underlying network (i.e., above signal of ≥ 0.5).

At higher signals, we found AUROC was saturated (approximately zero slope) inhibiting its ability to distinguish networks. AUPR and MCC both showed small variance across the levels of noise and limited saturation at both extremes. The high variability of FDR at low signal appeared not to be an issue even with very small sample sizes, as evidenced by the low iteration error bars in Figure 2 at sample size of 5. MCC was approximately linear with low variance across signal levels and some saturation at the highest levels (i.e., above > 0.9).

The aspirational network was also used to evaluate the impact of noise on the proposed centrality based measures. An ideal response to increasing signal would be a linear decrease in error. Supplementary Figure S2 shows the four centrality measures with increasing signal. Betweenness (Supplementary Figure S2A) had a discontinuity in response at relatively high signal strength (> 0.6) suggesting susceptibility to noise. Closeness (Supplementary Figure S2B) had an approximately flat response to increasing signal between 0.2 through 0.5 indicating it would be unable to differentiate noisy networks. Degree (Supplementary Figure S2C) had two modes; high error and high variance below signal 0.2, and approximately linear with low variance above this point. Empirically, the high error region is relevant when using a sample size of 5 (Figure 3C). PageRank (Supplementary Figure S2D) was considered to work well due to its nearly linear response beginning at a signal factor of 0.2. We also considered using eigenvector centrality, but found convergence issues due to disconnected subgraphs (data not shown).

PCLRC Algorithmic Limit

The probabalistic approach of $PCLRC$ was hypothesized to perform well at very low sample sizes due to resampling reducing the influence of outliers and we observed this for FDR in Figure 2, exemplified at sample sizes greater than 10. However, in the same figure we found that $PCLRC$ peaks for MCC with a sample size of 20, plateaued at a lower value with additional samples.

As defined in Saccenti et al [2015], $PCLRC$ is provided a set of samples from which to resample (with replacement). Each subset is used to infer a network and the top k% edges are kept. As the number of samples increases, the network inferred by $PCLRC$ converges. When the sample size is high, noise plays a smaller role and the strongest edges can be reliably kept. This causes weak but still meaningful edges to be discarded lowering the overall edge quality. When the sample size is low, high confidence edges can be found, and through the impact of noise, low edges can be observed. We show here that the peak at sample size 20 represented the balance between adding more samples for a better estimate of the underlying network and the overconfidence of $PCLRC$ in detecting important edges. To decouple these competing effects, we repeated $PCLRC$ methods allowing resampling from the full 500 samples. By resampling from the full set, we reduced the impact of the initial selection caused by sample size.

Supplementary Figure S3 shows evaluation metrics of our 'unlimited' $PCLRC$. Both the AUPR and AUROC plateau demonstrated that these performance measures are less impacted by small sample sizes. While consistent performance measure across samples sizes is desirable, neither reached a value indicating high quality network inference. FDR decreased as sample size increased. Unlike AUPR and AUROC, where sample size made little difference, the error in FDR appeared to be caused by estimating using an insufficient number of samples. $PCLRC_P$ showed a significantly faster decrease in FDR than the other methods, and $PCLRC_{MI}$ showed a significantly slower decrease. MCC presented a decrease in quality with additional samples, following our explanation above.

Network Differentiation

In the context of network differentiation, Supplementary Figure S4 shows inferred networks from two different metabolomic states. Using 200 samples for each of two states, networks were inferred using CLR_P. These inferred networks were overlaid on the reference network. For visual clarity, the names of metabolites are omitted. Both states resulted in disconnected graphs and both reliably identified the modified 15-PGDH enzyme interaction (the edge between the pairs of green nodes). However, the presence or absence of an edge cannot reliably be attributed to state difference or sampling noise, leading us to propose the bootstrap network method in section *Bootstrapped Networks*. This proposed bootstrap method for network differentiation relied on minimizing the sampling noise by repeatedly resampling subsets of the original data. Supplementary Figure S5 presents intermediate steps culminating in Figure 5. The left panels show how much sampling variation was observed when selecting a subset of 100 samples from the available 200 samples. These subsamples were used to create a single inferred network by selecting interactions that occur in $> 50\%$ of the inferred networks. The young and aged variations found few interactions that disagreed between resamplings (4 and 5, respectively). Due to the fact that these were undirected, the upper left triangle is used to list the interactions in all cases. The disagreement between the two states ultimately included 38 discordant interactions. The difference between these states include distant impacts that are more clearly visualized in Figure 5.

Full Resolution Images

Supplementary Figures 6-9 show pairwise and centrality evaluation results on all considered NIA methods. The corresponding figures in the paper show a sample of the NIAs to not distract from our primary message that NIAs fail to meaningfully converge to the underlying network (Figures 2 and 3, respectively).

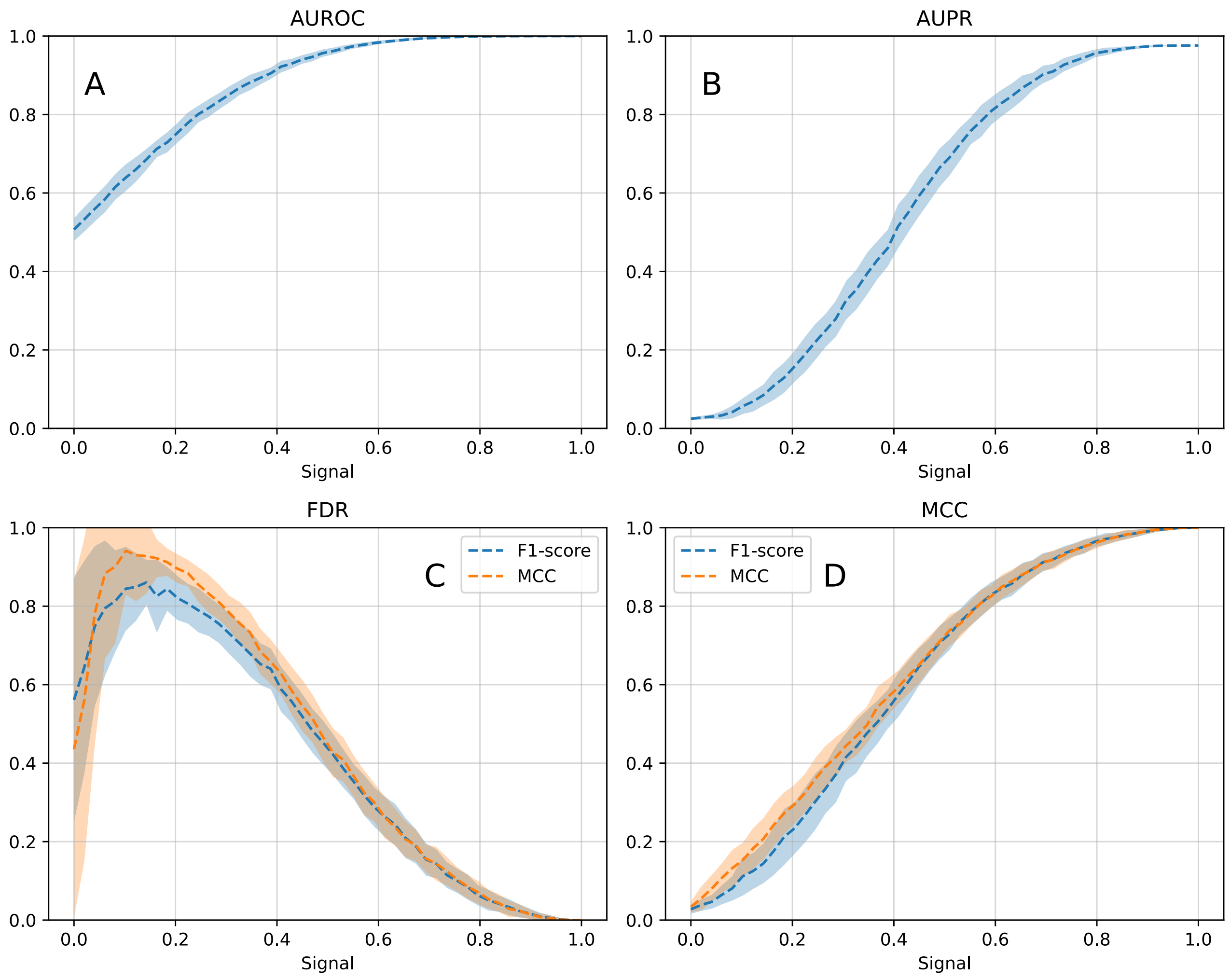

Supplementary Figure S1. Pairwise metric evaluations of an aspirational network. Networks were created using randomly generated noise added to the true adjacency matrix. Evaluation measures were calculated given the optimal thresholding value (calculated for each network). One hundred repetitions were performed for each of the 50 signal levels with dotted lines indicating mean value, and shaded region showing one standard deviation. A) Area Under Receiver Operating Curve (AUROC) variation. B) Area Under Precision Recall Curve (AUPR). Optimal threshold values for False Discovery Rate (C) and Matthews Correlation Coefficient (D) were calculated using both F1-score and MCC.

Paper Source

The work described in this article were originally explored in the Masters thesis by Haley Greenyer for her degree from the University of Victoria [Greenyer, 2022]. The thesis presents similar evaluation metric testing including the use of graph centrality measures, but does not consider the convergence issues of the network inference algorithms.

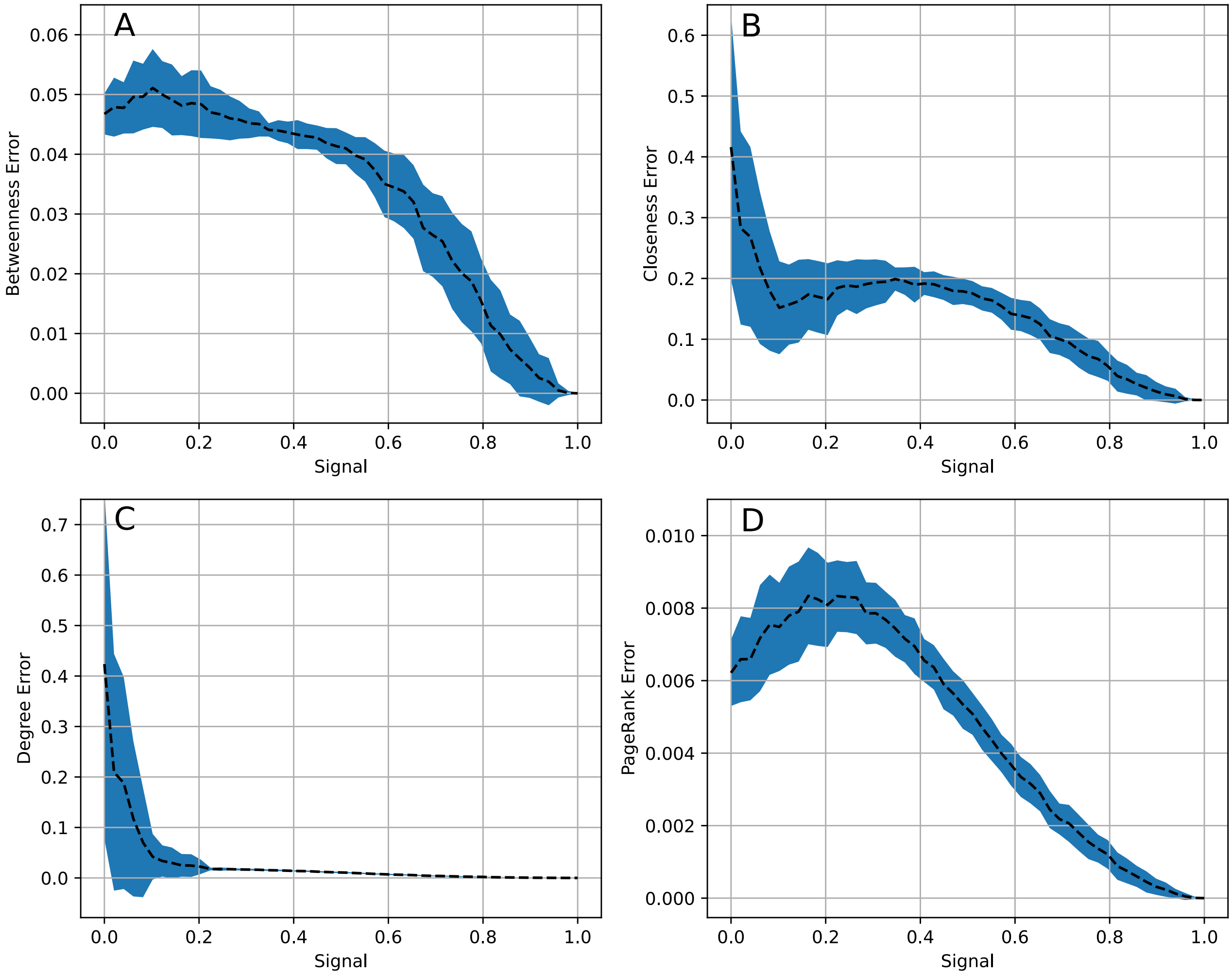

Supplementary Figure S2. Network Structure evaluations for an aspirational network. Networks were created using uniformly sampled noise and the reference adjacency matrix to test centrality-based evaluation metrics. Optimal threshold values were calculated using F1-score. Signal levels were repeated 100 times and in increments of 0.02. Dotted lines indicate mean value and the shaded region showing one standard deviation.

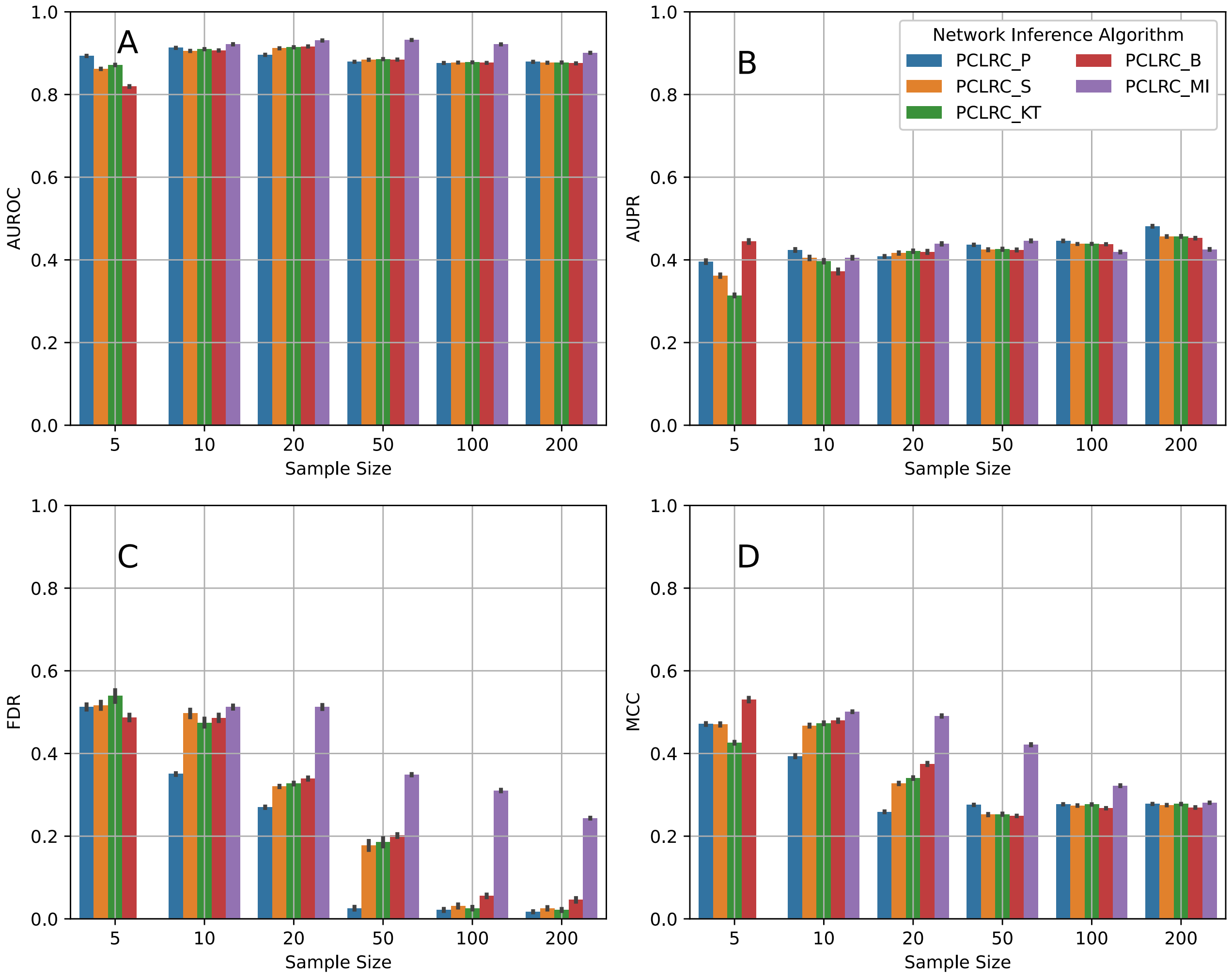

Supplementary Figure S3. $PCLRC$ pair-wise results when resampling from all 500 simulated sampled instead of a subset of the given size. Compare with Figure 2 where MCC peaks for $PCLRC$ methods at low sample sizes. Each method is replicated 100 times at each sample size.

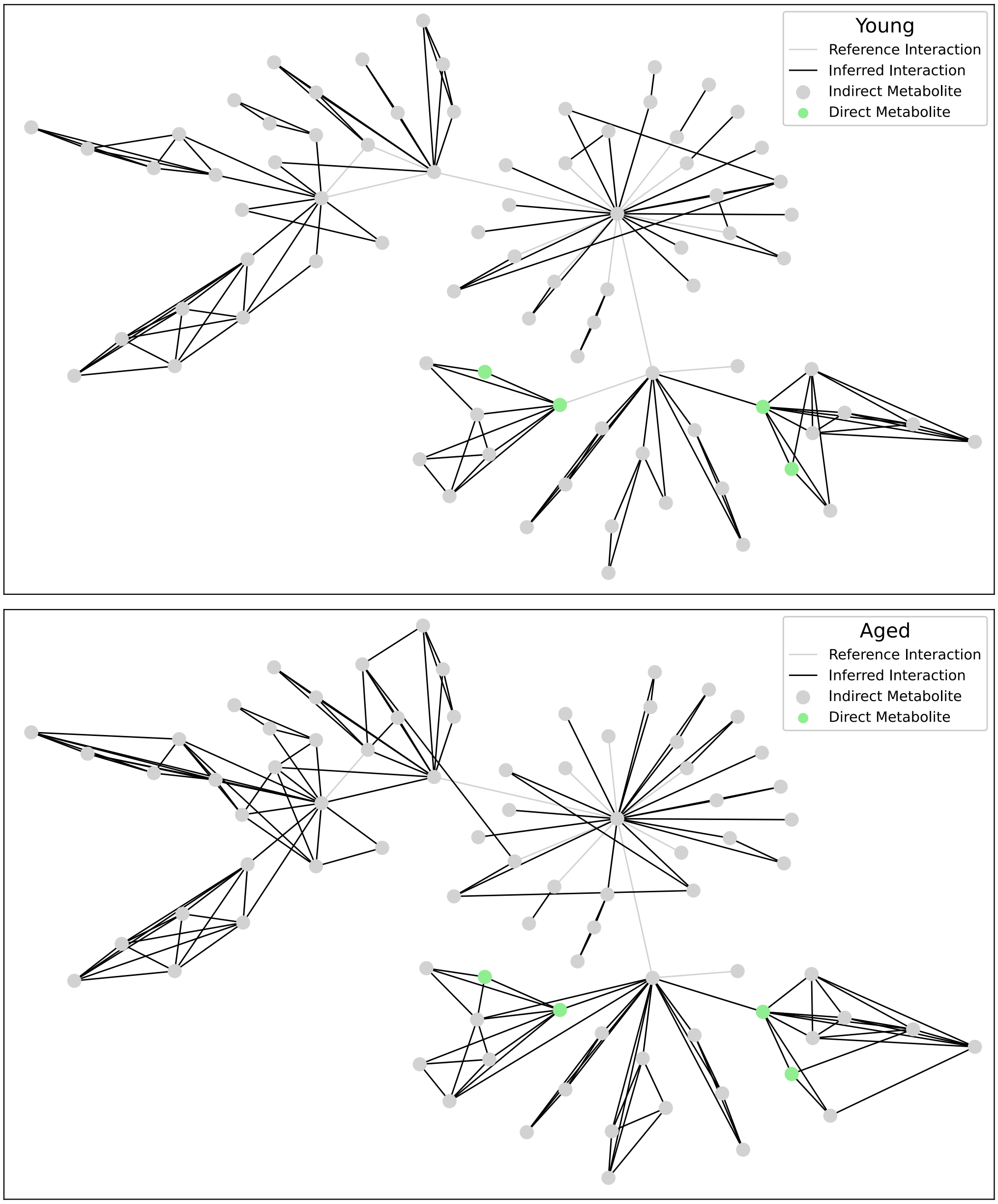

Supplementary Figure S4. A pair of inferred networks from young and aged datasets. Gray lines show ground-truth edges not found by the inference algorithm. The top graph was inferred from 200 samples from the young distribution of samples; the bottom from the aged distribution. Metabolites directly impacted by the mutation of 15-PGDH are shown in green.

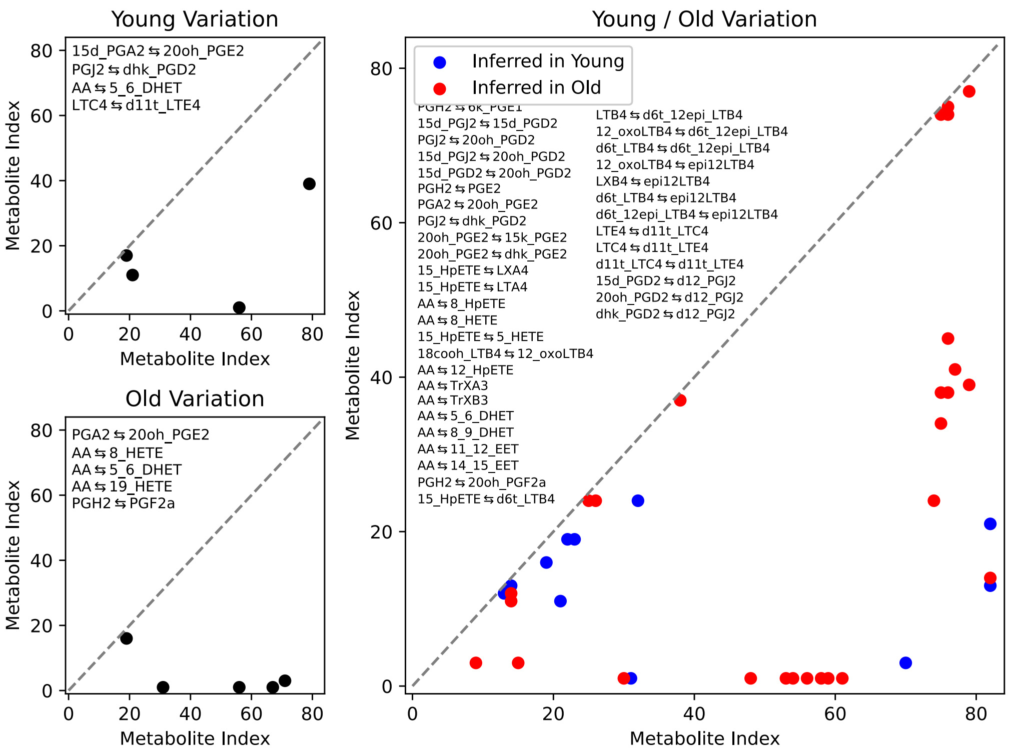

Supplementary Figure S5. Bootstrapped network differentiation. Young variation shows 4 edges were in disagreement when comparing two bootstrapped networks. Aged variation found 5 edges disagreed. Young/Aged variation shows 38 edges disagree when comparing a young and aged network. Edges that exclusively occur in young are shown in blue; edges exclusively in the aged network are in red. For all panels the reactions in disagreement between networks are written in the upper left.

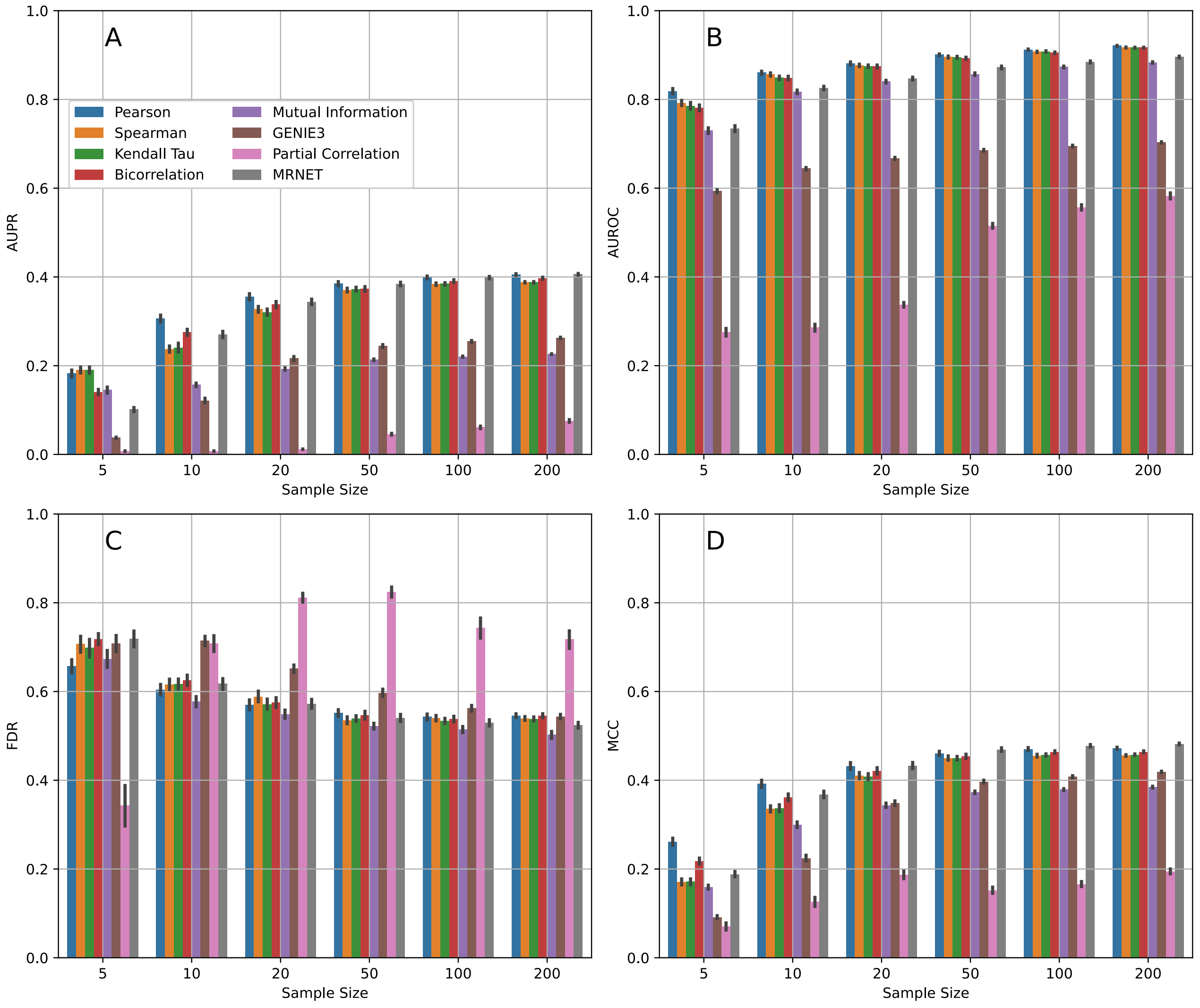

Supplementary Figure S6. Network inference algorithms (NIAs) evaluated on pairwise performance measures. Thresholding values for FDR and MCC were calculated optimizing for F1-score for each individual network to limit the effect of threshold selection. This selection of NIAs exclude CLR-based methods for visual clarity. See Figure S7 for CLR-based methods.

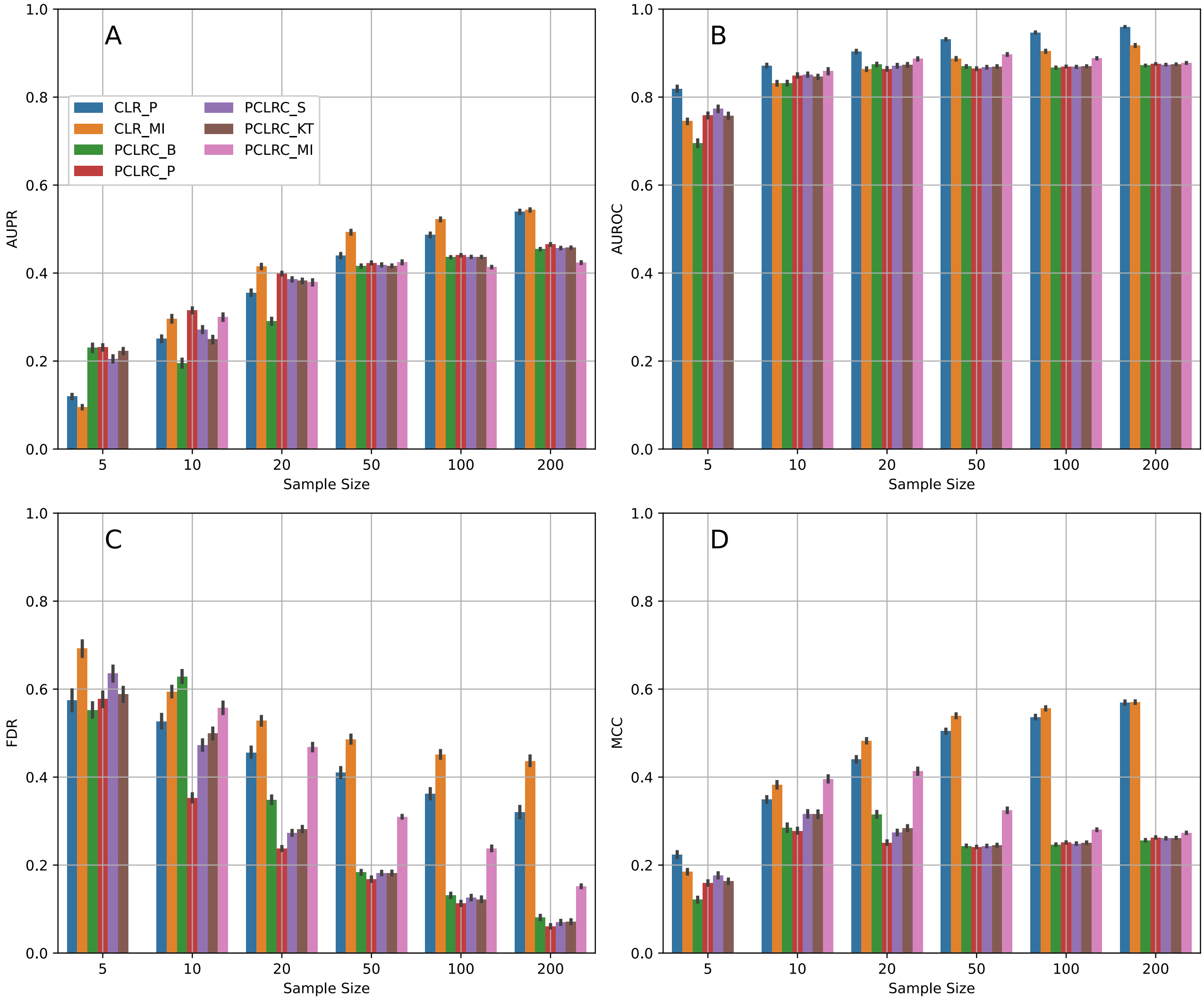

Supplementary Figure S7. Network inference algorithms (NIAs) evaluated on pairwise performance measures. Thresholding values for FDR and MCC were calculated optimizing for F1-score for each individual network to limit the effect of threshold selection. This selection of NIAs are CLR-based methods for visual clarity. See Figure S6 for non-CLR-based methods.

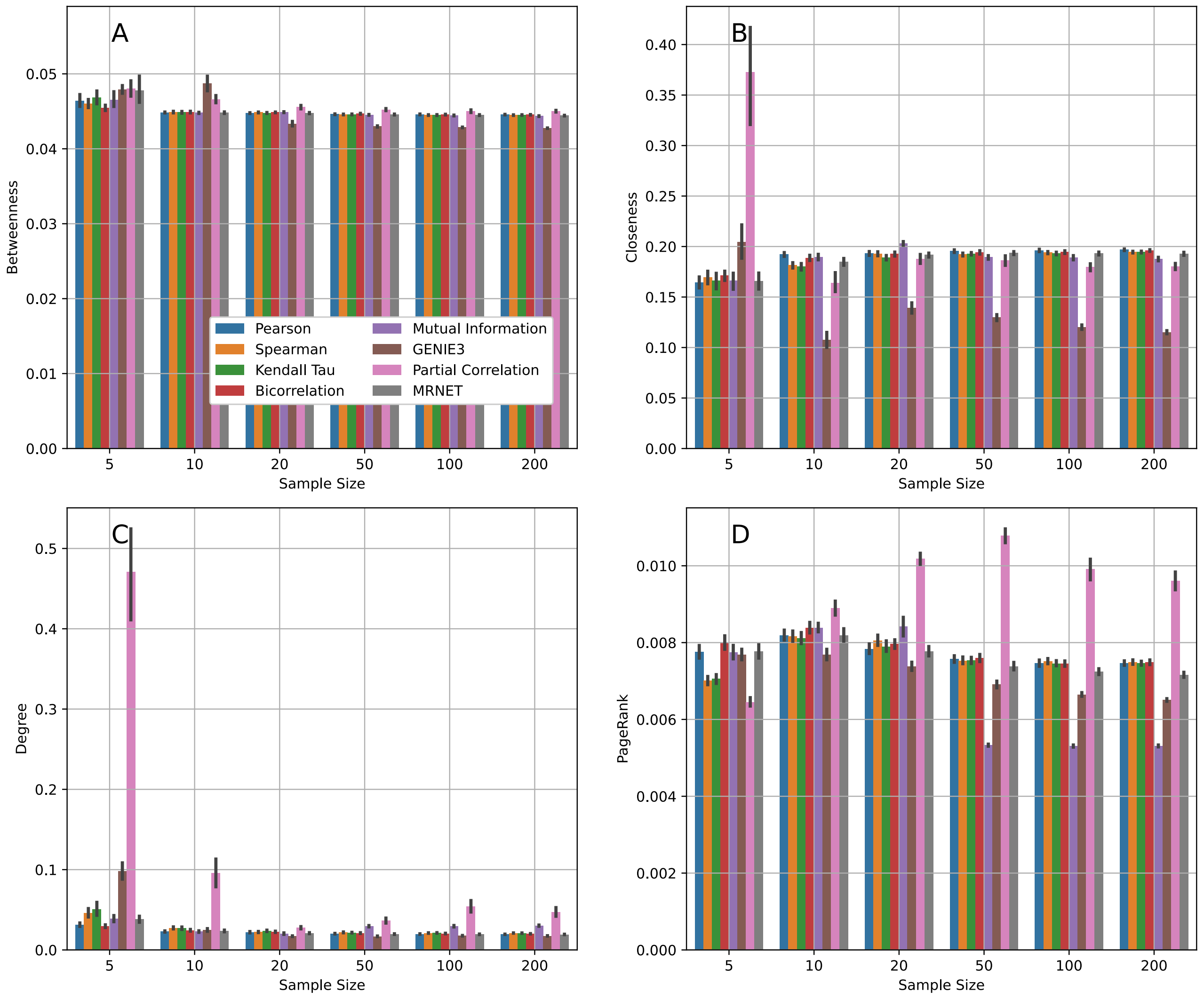

Supplementary Figure S8. Network inference algorithms (NIAs) evaluated on network-level performance measures. Thresholding values were calculated optimizing for F1-score for each individual network to limit the effect of threshold selection. This selection of NIAs exclude CLR-based methods for visual clarity. See Figure S9 for CLR-based methods.

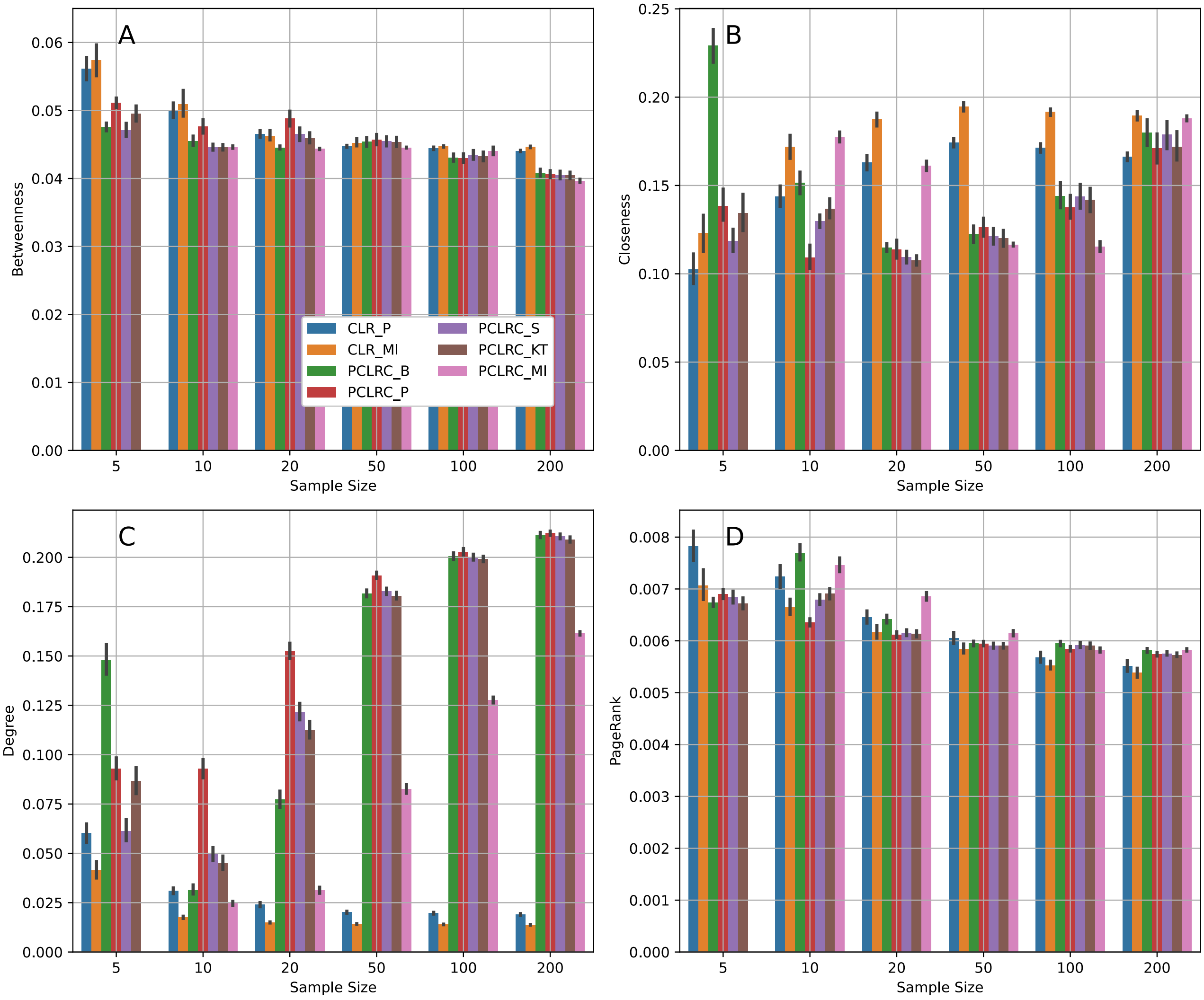

Supplementary Figure S9. Network inference algorithms (NIAs) evaluated on network-level performance measures. Thresholding values were calculated optimizing for F1-score for each individual network to limit the effect of threshold selection. This selection of NIAs are CLR-based methods for visual clarity. See Figure S8 for non-CLR-based methods.
